# Supplementary material for: Epigenetic dysregulation of naive CD4+ T-cell activation genes in childhood food allergy
Source: Nat Commun. 2018 Aug 17;9:3308. doi: 10.1038/s41467-018-05608-4 (PMC6098117; doi:10.1038/s41467-018-05608-4)
Supplement: Supplementary file 1 — Supplementary Information [file 41467_2018_5608_MOESM1_ESM.pdf]

Epigenetic dysregulation of naïve CD4+ T-cell activation genes in childhood food allergy

Authors: David Martino[1,4], Melanie Neeland[1], Thanh Dang[1], Joanna Cobb[1], Justine Ellis[1], Alice Barnett, Mimi Tang[1], Peter Vuillermin[1,2], Katrina Allen[1,*], Richard Saffery[1] and the HealthNuts investigators [+].

Affiliations:

[1]Murdoch Children's Research Institute, The University of Melbourne Department of Paediatrics, Flemington Road, Parkville, Victoria, 3052, Australia.

[2]Barwon Health, Geelong, Victoria, 3220, Australia.

[3]Deakin University, Geelong, Victoria, 3216, Australia.

[4]Telethon Kids Institute, 100 Roberts Road Perth, Western Australia.

[+]HealthNuts investigators who are not named authors: Shyamali Dharmage, Melissa Wake, Lyle Gurrin, Anne-Louise Ponsonby, Adrian Lowe.

*To whom correspondence should be addressed: katrina.allen@mcri.edu.au

Supplementary Table 1 - Clinical characteristics of study participants

	Controls (n=21)	Cases (n=45)	
		transient egg allergy (n=31)	*persistent egg allergy (n=13)*
**Demographics**			
SEIFA quintile (median)	3	3	3
Gender: males (n,%)	9 (43)	15 (48)	6(46)
Caucasian (n,%)	17 (81)	21 (68)	11 (85)
Family History FA (n,%)	0(0)	7(23)	1 (8)
Has siblings (n,%)	13 (62)	12 (39)	7 (54)
Attended day care (n,%)	8 (38)	9 (29)	3 (23)
Median age egg introduction (month)	5.5	6	5.5
Pet ownership (n,%)	10 (47)	12 (39)	7 (53)
**Clinical characteristics**			
Egg Allergy (12m) (n,%)	0	26 (100)	18 (100)
Egg Allergy (followup) (n,%)	NA	0 (0)	18(100)
Average SPT egg white (12m) (mm)	0.2	3.9	5.3
Average SPT egg white Followup (mm)	NA	0.75	3.2
Average sIgE egg white (kU/mL)	0.09	1.1	10.4
Average sIgE egg white Followup (kU/mL)	NA	0.44	7.9
Peanut Allergy (12m)	0 (0)	0 (0)	0 (0)
Peanut Allergy (followup)	0 (0)	0 (0)	0 (0)
Eczema diagnosis at 12m	7 (33)	13 (42)	7 (53)

SEIFA: socioeconomic index for areas; sIgE: specific Immunoglobulin E; SPT: skin prick test

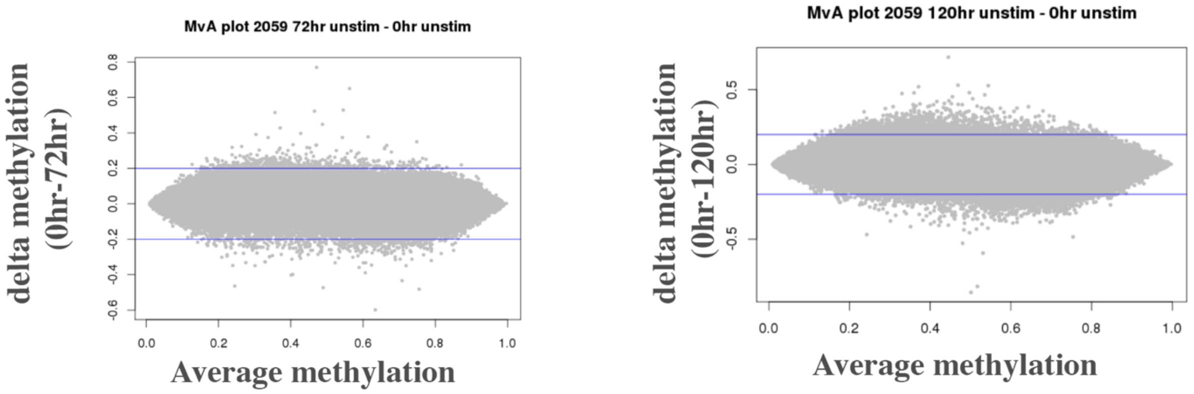

Supplementary Figure 1. Time course of culture-induced DNA methylation changes. Genome-wide DNA methylation data from naïve CD4+ T-cells isolated from two healthy infant donors. Methylation profiles were generated using the IlluminaHumanMethylation 450k array with 0hr, 72hr and 120 hr time points. MvA plots show the fold change (x-axis) as a function of average methylation (y-axis) representative data from one infant. Left panel is the change in DNA methylation between 0hr and 72hr unstimulated naive T cells, and right panel is 0 hr and 120 hr unstimulated naïve T cells. Blue lines are drawn at fold change +/- 20%. A substantially higher number of data points exhibited a fold change of more than 20% after resting in culture for 120 hours compared with 72 hours.

Supplementary Table 2- Test statistics for enrichment of TCR activation-associated differentially methylated CpG at histone modifications in naïve T-cells determined by ChIP-seq. ChIP-seq data were downloaded from the ENCODE consortium and signal peaks were tested for overlapping segments using Forbes coefficient.

#Rank	Track title	Similarity to query track	P-value	Overlap between query and reference track (bps)	Genome coverage of track (bps)	Nr. of reference track elements
1	E038-H3K4me1.broadPeak	6.36	0.004	452	394351178	185925
2	E038-H3K4me3.broadPeak	4.12	0.004	297	399524440	338126
3	E038-H3K27ac.broadPeak	3.08	0.004	298	536236329	425959
4	E038-H3K36me3.broadPeak	1.87	0.004	187	555781954	254336
5	E038-H3K9me3.broadPeak	0.58	0.669	82	778291280	509626
6	E038-H3K27me3.broadPeak	0.67	1.00	83	683853862	413197

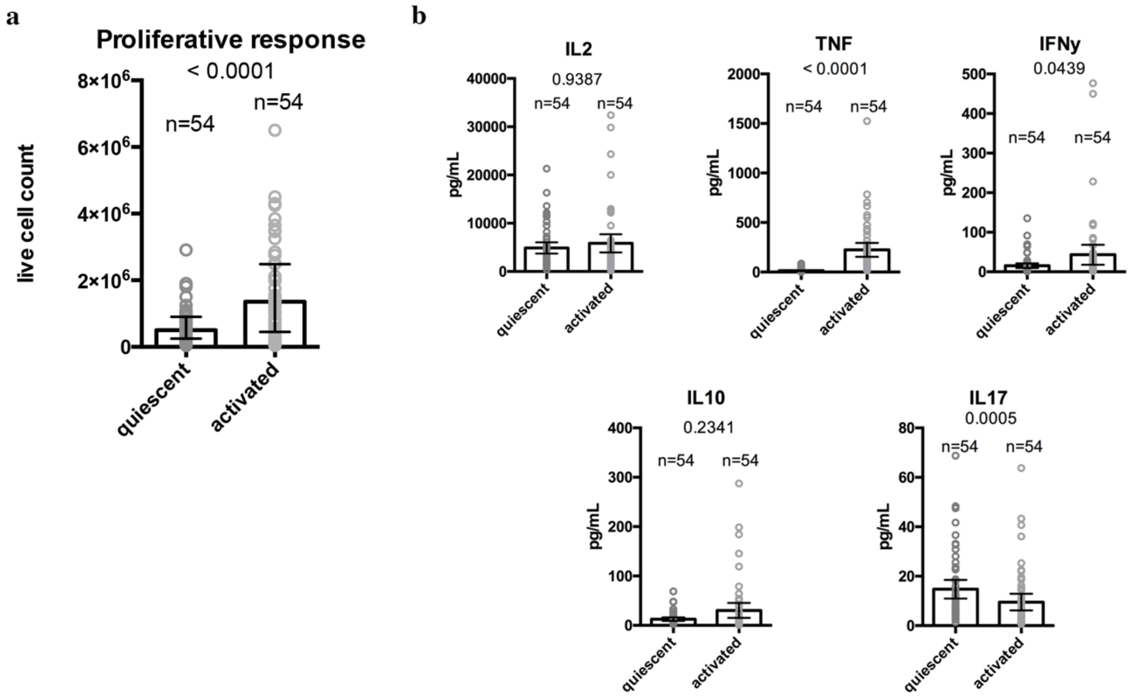

Supplementary Figure 2. Function responses to anti-CD3/CD28 stimulation at 12-months. Following 72 hours of stimulation, activated and quiescent cells were harvested and counted using an automated cell counter. **(a)** Proliferative response is presented as absolute cell counts. **(b)** Cell supernatants were assayed for a range of cytokines using multiplexed bead arrays. Activation of T-cell induced significant induction of TNF and IFNy. IL4 and IL6 produciton was below detection limits (data not shown). All data are presented as median with interquartile range, groups compared using the Mann Whitney test for matched samples. Exact P values are shown.

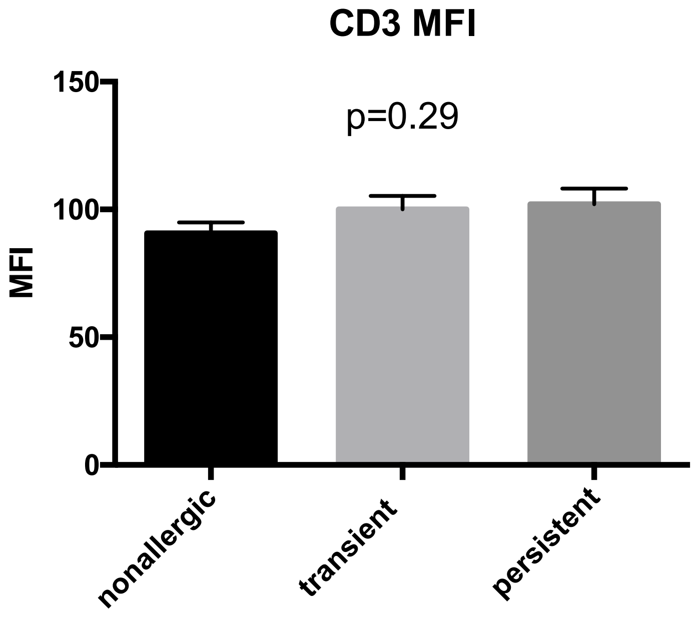

Supplementary Figure 3. – Surface expression of CD3 on naïve T-cell collected at baseline. Graph shows the mean fluorescence intensity (MFI) for each group. Data is presented as mean with standard error. Groups were compared using a one-way ANOVA.

Supplementary Table 3- differentially expressed and methylated genes in quiescent naïve T cells

Differentially expressed genes

Ensmbl ID	symbol	biotype	description	logFC	AveExpr	t	P.Value	adj.P.Val
ENSG00000080854	IGSF9B	protein_coding	immunoglobulin superfamily, member 9B	0.72	4.20	4.98	0.00	0.04
ENSG00000112167	SAYSD1	protein_coding	SAYSVFN motif domain containing 1	0.31	4.12	4.94	0.00	0.04

Differentially methylated CpG

Probe ID	symbol	chr	position	deltabeta	AveMeth	t	P.Value	adj.P.Val
cg26475005	KIAA1217	chr10	24649480	-0.03	-0.61	-6.24	0.00	0.01
cg25316898	C18orf21	chr18	33552869	0.01	-3.28	5.71	0.00	0.04

Ave.Expr = average expression level, Adj.P.Val = false discovery rate corrected P value, chr = chromosome, deltabeta = absolute difference in methylation (case – control), logFC = log fold change (cases – control), pos = position.

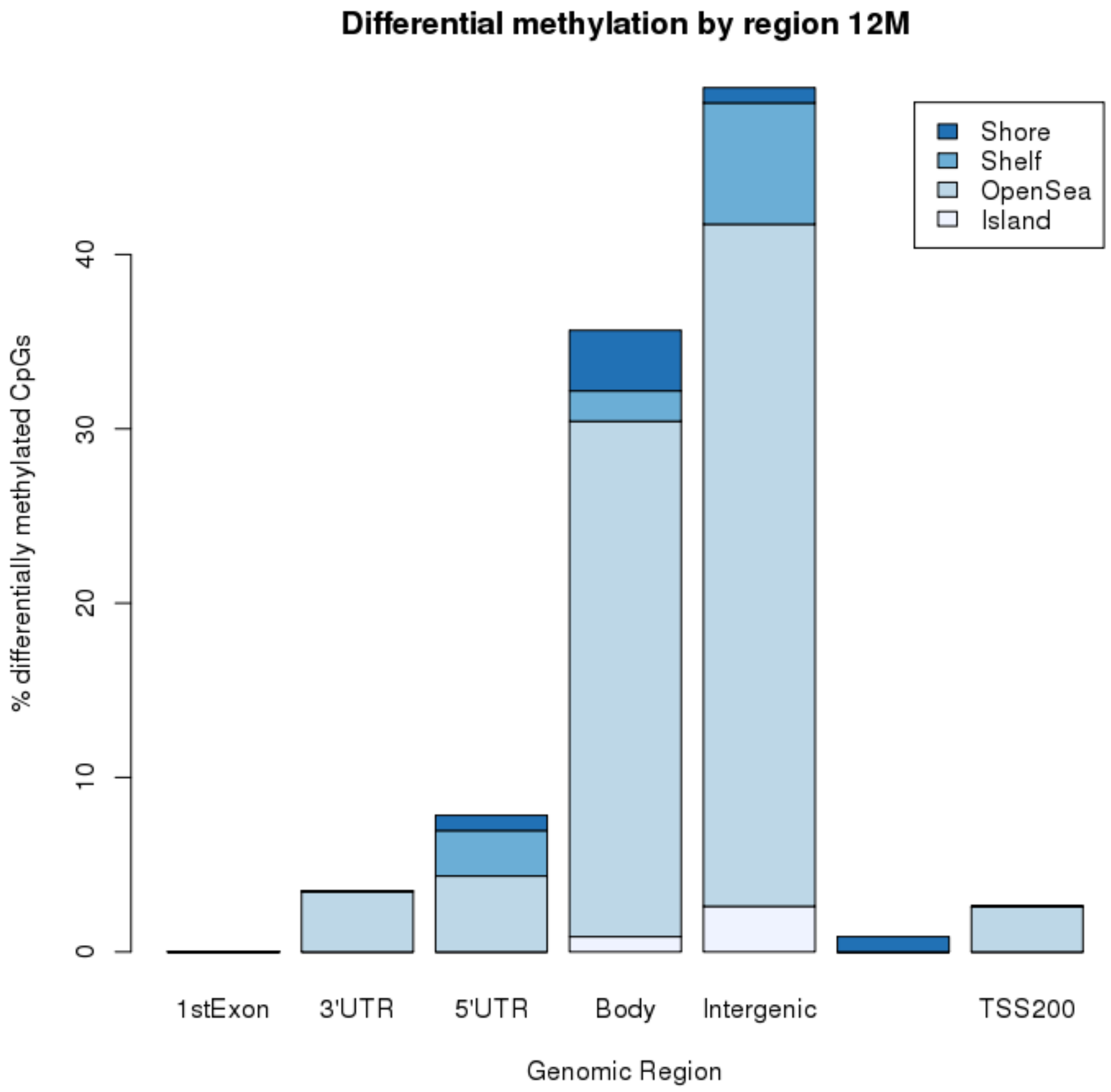

Supplementary Figure 4 – Overlap of differentially methylated CpG with genomic regions. Bar chart shows the percentage of food allergy associated differentially methylated regions stratified by genomic feature.

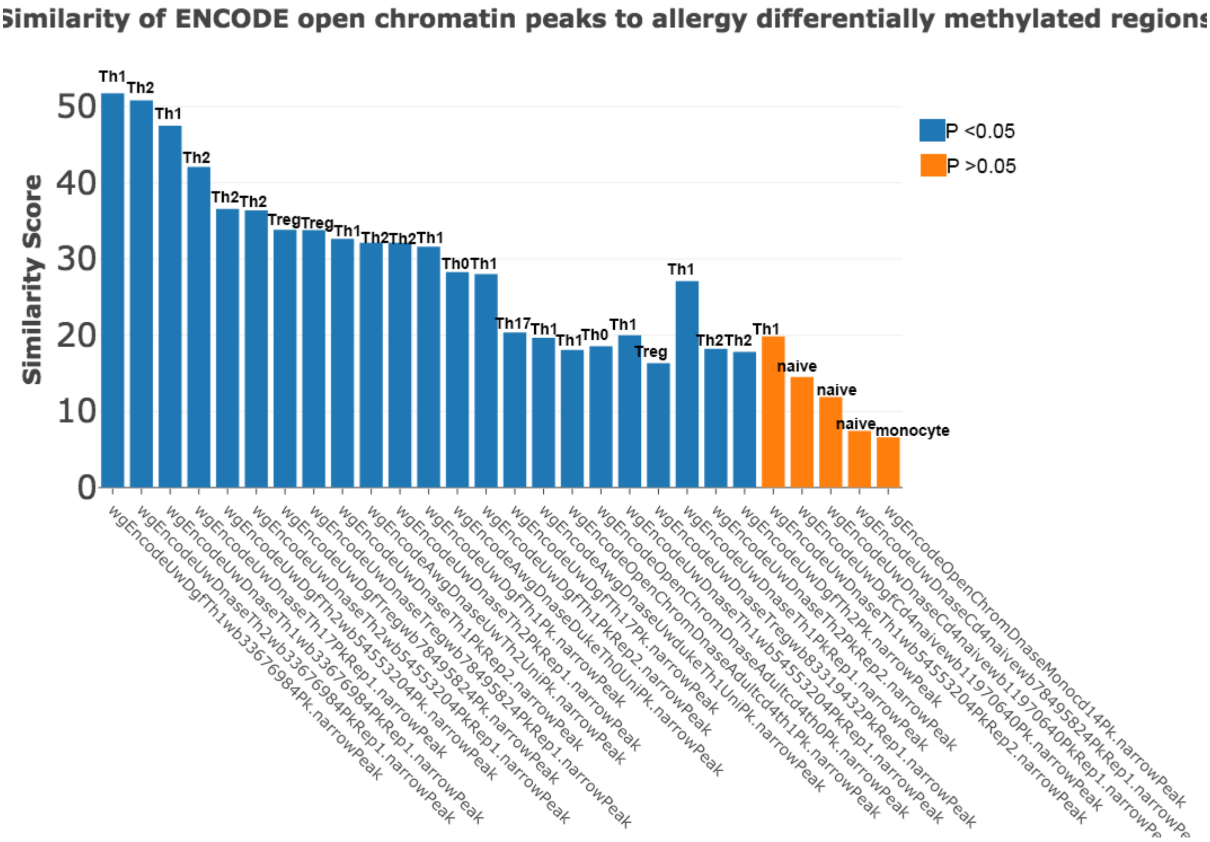

Supplementary Figure 5 – Enrichment test of allergy dmrs compared to effector, naïve and monocyte open chromatin regions. Publicly available digital genomic footprint and DNAse I peaks for differentiated effector CD4 T-cells (Th1, Th2, Th17, Treg), naïve T-cells and monocytes were downloaded from the ENCODE consortium data repository. A statistical test was performed comparing overlaps of allergy-dmrs to ENCODE DNAse1 and footprint peaks using the Forbes coefficient, and the datasets were ranked in order of descending similarity to allergy dmrs (y-axis). Significantly enriched open chromatin peaks are coloured blue, and p-values were determine using monte carlo randomization strategy. Exact data track names are shown on the x-axis. Allergy-dmrs were more enriched in regions of accessible chromatin that define effector T-cells rather than naïve T-cells. Data from monocytes was included as a negative control.

Supplementary Table 4 – Association analysis of candidate SNPs with food allergy. SNPs showing weak evidence for an association with food allergy are highlighted. Analysis is based on n=42 under a logistic model adjusted for ancestry.

CHR	SNP	BP	GENE	A1	NMISS	OR	STAT	P
1	rs4240895	9713386	PIK3CD	T	42	1.47	0.73	0.464
1	rs57784284	9716125	PIK3CD	A	41	1.58	0.71	0.476
1	rs35478578	66321311	PDE4B	A	42	0.77	-0.33	0.745
1	rs17451451	66388153	PDE4B	G	42	0.47	-0.97	0.332
1	rs2310752	66392405	PDE4B	A	42	0.97	-0.05	0.962
1	rs12096433	66430203	PDE4B	A	41	0.35	-0.84	0.399
1	rs2503215	66490013	PDE4B	A	42	0.63	-0.60	0.545
1	rs74969060	66490092	PDE4B	G	41	0.61	-0.63	0.526
1	rs7530150	66513674	PDE4B	A	41	0.54	-0.65	0.515
1	rs76165263	66587178	PDE4B	A	41	NA	NA	NA
1	rs11208807	66634921	PDE4B	T	42	0.53	-1.10	0.271
1	rs6689595	66776137	PDE4B	C	40	0.34	-1.64	0.102
1	rs61796507	66837253	PDE4B	T	41	0.17	-1.54	0.123
1	rs6667969	66849760	PDE4B	T	42	2.14	1.39	0.164
1	rs57569414	154380419	IL6R	A	42	0.69	-0.44	0.657
1	rs4537545	154418879	IL6R	T	42	0.68	-0.68	0.497
2	rs13401241	25518470	DNMT3A	C	42	0.93	-0.13	0.893
2	rs6711622	25531350	DNMT3A	A	42	0.93	-0.15	0.883
2	rs7575625	25548902	DNMT3A	G	42	0.66	-0.78	0.436
2	rs10203724	102688158	IL1R1	A	42	1.15	0.18	0.855
2	rs12712126	102696472	IL1R1	C	41	2.40	1.51	0.132
2	rs13029804	102723017	IL1R1	C	42	0.61	-0.79	0.427
2	rs7580384	102760718	IL1R1	A	42	1.99	1.18	0.240
2	rs1420106	103035044	IL189RAP*	T	41	1.63	0.73	0.464
2	rs2058659	103054556	IL189RAP*	T	42	1.14	0.22	0.826
2	rs11465702	103057892	IL189RAP*	G	42	1.80	0.74	0.457
2	rs10166061	238906511	UBEF2-SLCY	C	41	1.08	0.09	0.926
2	rs4663826	238908120	UBEF2-SLCY	T	42	1.13	0.15	0.877
3	rs73228187	111308568	CD96	G	42	1.10	0.12	0.904
3	rs17281304	111323019	CD96	T	42	2.93	1.04	0.300
3	rs73230114	111328598	CD96	A	42	0.74	-0.40	0.690
3	rs9868266	111338169	CD96	T	42	1.07	0.08	0.934
11	rs80226633	44588256	CD82	C	41	NA	NA	NA
5	rs803139	132131024	IL4	A	41	1.53	-0.29	0.444
6	rs6937545	32418031	HLADQB1	A	42	0.8	-0.291	0.771
7	rs6946141	24925834	OSBPL3	G	42	0.56	-1.15	0.250

11	rs11038069	44605335	CD82	A	42	1.04	0.08	0.939
11	rs3781753	44630049	CD82	A	41	0.99	-0.02	0.980
11	rs80053099	44642255	CD82	C	42	2.61	1.36	0.173
13	rs55708275	41174224	FOXO1	A	42	0.75	-0.44	0.662
13	rs1986649	41178824	FOXO1	T	42	0.52	-0.84	0.403
13	rs4429172	41189143	FOXO1	A	42	0.58	-1.00	0.319
13	rs7335520	41196407	FOXO1	T	42	0.56	-1.07	0.287
13	rs56204202	41234397	FOXO1	C	41	0.87	-0.18	0.856
17	rs4889856	78513632	RPTOR	C	41	1.82	1.12	0.261
17	rs11651818	78530300	RPTOR	C	42	0.25	-1.66	0.096
17	rs12942701	78544604	RPTOR	A	41	0.60	-0.57	0.565
17	rs7226296	78547657	RPTOR	G	41	0.71	-0.62	0.534
17	rs7215496	78547753	RPTOR	T	42	1.91	0.94	0.346
17	rs12951309	78558286	RPTOR	A	42	1.41	0.46	0.646
17	rs7225476	78561603	RPTOR	G	42	0.37	-1.56	0.120
17	rs201324621	78601169	RPTOR	A	42	1.41	0.46	0.646
17	rs8072592	78615361	RPTOR	G	42	0.42	-1.24	0.215
17	rs9906827	78665405	RPTOR	T	42	3.33	1.81	0.070
17	rs1564868	78685176	RPTOR	G	42	0.66	-0.73	0.464
17	rs9915378	78708418	RPTOR	A	40	0.62	-0.85	0.395
17	rs6565479	78712423	RPTOR	A	42	1.08	0.13	0.896
17	rs72852717	78741954	RPTOR	A	39	NA	NA	NA
17	rs7225755	78761708	RPTOR	C	42	0.82	-0.38	0.707
17	rs72852775	78761726	RPTOR	G	42	0.27	-1.22	0.224
17	rs2672886	78781651	RPTOR	C	41	4.20	1.99	0.047
17	rs734338	78782340	RPTOR	A	42	1.66	1.05	0.295
17	rs73351957	78829069	RPTOR	A	42	4.00	0.93	0.351
17	rs9912092	78841693	RPTOR	G	42	0.71	-0.52	0.605
17	rs2289764	78865546	RPTOR	C	42	1.21	0.30	0.763
17	rs9908768	78902775	RPTOR	A	41	9.20	1.68	0.093
18	rs12964682	60786227	BLC2	G	42	0.66	-0.79	0.431
18	rs956572	60820571	BLC2	A	42	0.85	-0.28	0.780
18	rs7230970	60821076	BLC2	C	41	0.70	-0.54	0.587
18	rs72943023	60841614	BLC2	G	42	0.75	-0.40	0.693
18	rs4941187	60849216	BLC2	C	42	1.49	0.78	0.435
18	rs12457700	60860246	BLC2	T	42	1.49	0.78	0.435
18	rs11152372	60860930	BLC2	G	42	1.33	0.56	0.577
18	rs17070848	60905500	BLC2	T	40	0.23	-1.41	0.159
18	rs7240326	60917351	BLC2	T	41	1.37	0.68	0.494
18	rs2849382	60976193	BLC2	C	42	0.38	-1.39	0.164
18	rs949037	60979013	BLC2	C	42	1.33	0.51	0.611

18	rs76945431	60989414	BLC2	T	42	NA	NA	NA
19	rs373658	3143320	GNA15	T	41	1.03	0.06	0.951
19	rs675975	3144508	GNA15	A	42	0.88	-0.25	0.803
19	rs35047169	3154978	GNA15	A	42	0.44	-0.64	0.523
20	rs2424904	31352316	DNMT3B	C	42	1.25	0.39	0.695
20	rs242542	31403426	DNMT3B	G	42	1.91	0.72	0.472
22	rs7290469	22150982	MAPK1	A	42	1.33	0.52	0.600
22	rs2070505	22162257	MAPK1	C	42	1.13	0.21	0.833
22	rs58212445	22209190	MAPK1	C	41	1.03	0.02	0.982
22	rs55762115	22224598	MAPK1	G	39	1.83	0.98	0.329

A1: minor allele, A2: major allele, F_A: frequency of minor allele in cases, F_U: frequency of minor allele in controls, BP: base position.

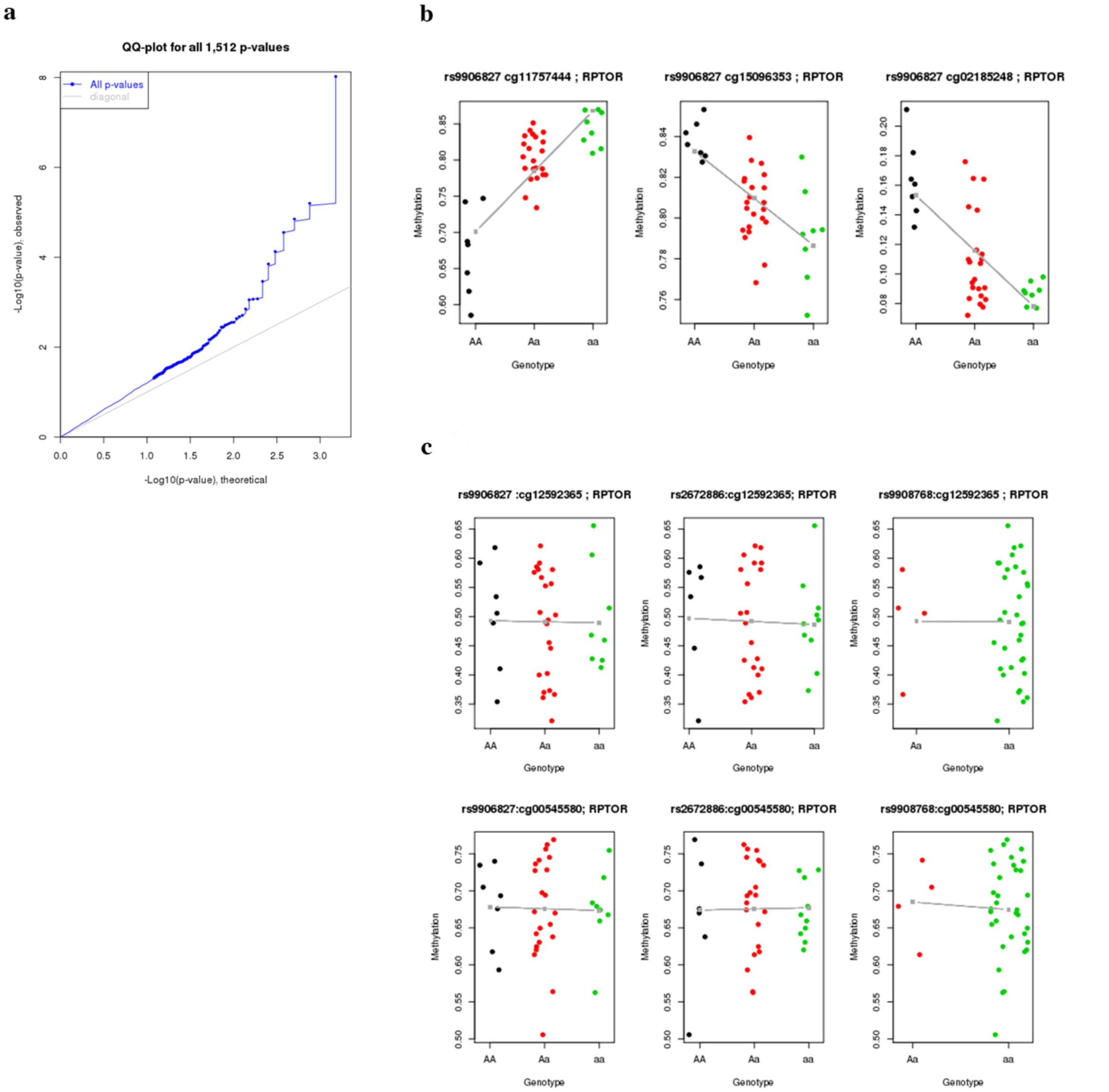

Supplementary Figure 6 – QQplots for QTL association analysis. (a) show the distribution of p-values for pairwise comparisons of SNPs with corresponding CpG methylation levels at *RPTOR*. (b) An example of significant *RPTOR* meQTL associations. (c) Boxplots for SNP: *RPTOR* pairs restricted to the food allergy-associated dmps in *RPTOR* (cg12592365, cg00545580)

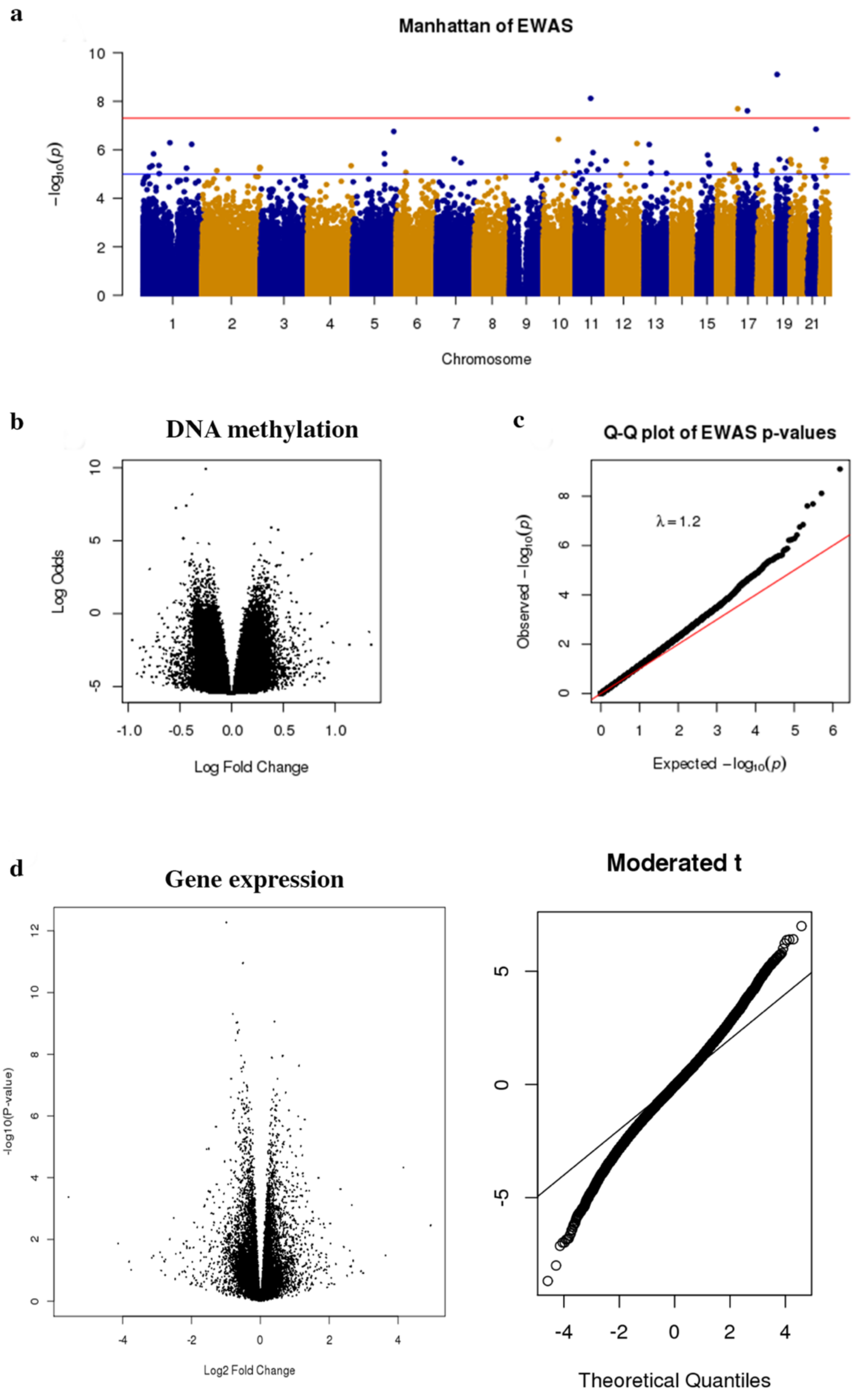

Supplementary Figure 7 – Diagnostic plots of genome-wide data modelling at baseline in activated cells. (a) Manhattan of epigenome-wide association (DNA methylation data). (b) Volcano plot of effect sizes. (c) qqplot of pvalue distribution with genomic inflation factor. (d) Volcanoplot and qqplot of moderated t statistic for RNAseq analysis.
