# Supplementary material for: Epigenetic dysregulation of naive CD4+ T-cell activation genes in childhood food allergy
Source: Nat Commun. 2018 Aug 17;9:3308. doi: 10.1038/s41467-018-05608-4 (PMC6098117; doi:10.1038/s41467-018-05608-4)
Supplement: Supplementary file 3 — Description of Additional Supplementary Files [file 41467_2018_5608_MOESM3_ESM.pdf]

Description of Additional Supplementary Files

File Name: Supplementary Data 1
Description: Differentially expressed genes in activated T cells

File Name: Supplementary Data 2
Description: Differentially methylated loci in activated T cells

File Name: Supplementary Data 3
Description: Differentially expressed genes in food allergy

File Name: Supplementary Data 4
Description: Differentially methylated loci in food allergy
